# Beating stress: music with monaural beats reduces anxiety and improves mood in a non-clinical population

**DOI:** 10.3389/fpsyg.2025.1539823

**Published:** 2025-06-18

**Authors:** Tara Venkatesan, Andrew Demetriou, Hendrik Vincent Koops, Daniel L. Bowling

**Affiliations:** ^1^Universal Music Group, London, United Kingdom; ^2^School of Advanced Study, University of London, London, United Kingdom; ^3^Department of Intelligent Systems, Delft University of Technology, Delft, Netherlands; ^4^Department of Psychiatry and Behavioral Sciences, Stanford University School of Medicine, Stanford, CA, United States; ^5^Center for Computer Research in Music and Acoustics (CCRMA), Stanford University, Stanford, CA, United States

**Keywords:** auditory beats, monoaural beats, music, anxiety, affect

## Abstract

Auditory beat stimulation in the delta–theta frequency range (0–7 Hz) is gaining interest as a non-invasive intervention for anxiety. This study investigated the effects of a relatively understudied form—monaural beats—and whether they produce acute changes in anxiety and mood when presented alone or embedded harmonically within music. Participants (*n* = 308) were randomly assigned to one of three 30-min listening conditions: (1) Monaural Beats + Music, (2) Monaural Beats-Only, or (3) a Pure Tone Control. Psychological effects were assessed via changes in self-reported anxiety (State–Trait Anxiety Inventory, state subscale) and mood (bipolar Likert scales for emotional valence, arousal, and energy). The results showed that only the Beats + Music condition significantly reduced anxiety from before to after listening with a medium effect size anxiety from before to after listening (*p* < 0.001, *d* = −0.58). Furthermore, only the Beats + Music significantly increased emotional valence from before to after listening (*p* < 0.001, *d* = 0.48). Finally, the Beats-Only condition showed a significant reduction in energy from before to after listening (*p* < 0.05, *d* = −0.28). These findings indicate that monaural beats can be harmoniously integrated into music without diminishing the anxiolytic properties of the latter, whereas presentation of beats alone has different effects. This suggests that integrating monaural beats within music may be a viable approach for targeted auditory neuromodulation.

## Introduction

### Definitions

Auditory beats are amplitude fluctuations that arise when two tones with similar fundamental frequencies (F0s) interact, producing alternating constructive and destructive interference at a rate equal to their frequency difference (e.g., 100 Hz and 104 Hz tones yield a 4 Hz beat). Beats are perceptible when F0s differ by ~1–40 Hz, depending on center frequency (Schwarz and Taylor, 2005). Smaller differences are heard as steady pulsations; larger ones elicit a sensation of “roughness” (Pratt et al., 2010; Oster, 1973; Schwarz and Taylor, 2005; von Helmholtz, 1863). Fluctuation depth, determined by the relative amplitudes of the tones, affects perceived salience (Grose et al., 2012). Monaural beats occur when both tones are played to both ears, creating physical amplitude modulation, while binaural beats arise when tones are presented separately to each ear, with the beat percept generated in the brain (Dove, 1844).

### History

Monaural beats have long been used in Tibetan and Indian monastic traditions—for example, in singing bowls supporting meditation and healing practices (Seetharaman et al., 2024). Scientific interest grew in 19th century Germany with Dove’s discovery of binaural beats (Dove, 1844) and Helmholtz’s recognition of monaural beating in music (von Helmholtz, 1863). Helmholtz viewed high-rate beats (~30–50 Hz) as dissonant irritants. Lower-rate beats, like those used in traditional contexts, received little attention until Oster (1973) *Scientific American* article revived popular interest in the topic (Oster, 1973). More recent research using EEG has shown that auditory beat stimulation can modulate neural oscillations (e.g., Dos Anjos et al., 2024; Draganova et al., 2008; Derner et al., 2021; Schwarz and Taylor, 2005), supporting the hypothesis that beats may promote neural entrainment with therapeutic relevance (Aarts et al., 2014; Carvajal et al., 2024; Stupacher et al., 2016; Stupacher et al., 2017).

### Therapeutic effects

Growing evidence suggests that listening to binaural beats in the delta (<4 Hz) and theta (4–8 Hz) frequency range can reduce physiological stress markers (e.g., heart rate variability, blood pressure) and self-reported anxiety (Demirci and Sezer, 2024; Isik et al., 2017; Kelton et al., 2021; Klimesch, 1999; Le Scouarnec et al., 2001; Loong et al., 2022; McConnell et al., 2014; Padmanabhan et al., 2005; Ölçücü et al., 2021; Wiwatwongwana et al., 2016). For example, Wiwatwongwana et al. (2016) found that 60 min of binaural beats combined with music before and during cataract surgery significantly reduced self-reported anxiety, blood pressure, and heart rate compared to a silent control condition (earphones without sound); heart rate was also lower than in a music-only control condition. Similar effects have been reported with shorter exposures (e.g., 10–30 min) and when beats are only presented preoperatively (Padmanabhan et al., 2005; Ölçücü et al., 2021). Outside clinical contexts, binaural beats have been linked to greater relaxation and cardiovascular recovery after-exercise (McConnell et al., 2014), as well as acute reductions in anxiety. In an online study, Mallik and Russo (2022) found that beats combined with music reduced anxiety—particularly cognitive symptoms like rumination—more effectively than music alone, beats alone, or pink noise in individuals with moderate trait anxiety (Mallik and Russo, 2022). Supporting the growth of this use case, a 2022 global survey found that 5.3% of the 30,896 respondents reported using binaural beats as a “digital drug” for stress relief (Barratt et al., 2022). Finally, although most studies have focused on binaural beats, the traditional use of monaural beats, along with evidence that they (1) elicit more salient percepts at equivalent beat frequencies and modulation depths (Oster, 1973), and (2) elicit stronger auditory steady-state responses in EEG recordings (Grose et al., 2012; Kuwada et al., 1984; Perez et al., 2020; Pratt et al., 2010; Schwarz and Taylor, 2005), suggests that monaural beats have greater neurophysiological and thus potentially stronger therapeutic effects on anxiety.

### Beats alone or embedded in music?

Many studies examining auditory beats embed the stimulus within other audio—e.g., pink noise (McConnell et al., 2014; Wahbeh et al., 2007), natural soundscapes (Wiwatwongwana et al., 2016; Kuratomo et al., 2019; Weiland et al., 2011; Shepherd et al., 2023), or music (Mallik and Russo, 2022; Padmanabhan et al., 2005; Demirci and Sezer, 2024; Ölçücü et al., 2021; Parodi et al., 2021; Le Scouarnec et al., 2001; Bhusari et al., 2023; Salehabadi et al., 2024; Wiwatwongwana et al., 2016)—possibly leveraging the anxiolytic effects of these forms (e.g., Krumhansl, 1997; Jiang et al., 2013; Sandstrom and Russo, 2010; Smith and Morris, 1977; Sokhadze, 2007). However, a meta-analysis of 22 studies examining the effects of beats on anxiety, analgesia, memory, and attention found that effects were stronger for beats alone than for beats embedded in music, raising the possibility that musical elements (e.g., melody, harmony, rhythm) may interfere with beat processing (Garcia-Argibay et al., 2019). That said, this conclusion is weakened by substantial methodological variability across studies. Embedding practices vary widely—some render beats nearly subliminal (e.g., Wiwatwongwana et al., 2016; Bang et al., 2019), while others present them prominently (e.g., Padmanabhan et al., 2005). Key parameters of beat stimuli, such as center frequency, modulation depth, and amplitude relative to the embedding stimulus, are often unreported (e.g., Bhusari et al., 2023), hampering cross-study comparisons, especially with respect to the question of which methods are best for beat stimulation. As a result, whether musical embedding enhances or interferes with beat-driven anxiolysis remains unresolved.

### The present study

To investigate the anxiolytic and mood elevating potential of auditory beat stimulation embedded in music, we conducted a single-blind randomized controlled trial comparing the effects of listening to Monaural beats embedded in music (Beats + Music), Monaural beats alone (Beats–Only), and a Pure Tone Control. The intervention lasted 30 min and was administered to a non-clinical online sample (N = 308). We hypothesized that music embedded with monaural beats—designed to be harmonically and rhythmically congruent with the music—would lead to greater reductions in state anxiety and improvements in mood than either Beats–Only or the Pure Tone Control.

## Methods

### Participants

Participants were recruited for this study from Prolific^1^, an online crowdsourcing platform for behavioral research with vetted participants and demographic pre-screening capabilities. All participants were based in the United States, had normal hearing, no self-reported psychiatric conditions, and were not currently taking medication for depression or anxiety. An *a priori* power analysis based on an expected medium effect size between the Beats + Music and Pure Tone Control conditions (Cohen’s *d* = 0.49; Mallik and Russo, 2022) indicated that a sample size of approximately 91 participants per condition would be required to achieve 95% power at an alpha level of 0.05. Given differences in our experimental design, and a projected attrition rate of 20% (e.g., due to failure to pass a headphone screener), we recruited 150 participants per condition (450 total) to ensure that the study would be adequately powered. Participants were paid at Prolific’s recommended hourly rate of $12.00. All participants provided informed consent. Our study protocol was approved by Salus, a third-party Institutional Review Board (protocol number 24387).

### Stimuli

**Figure 1:**
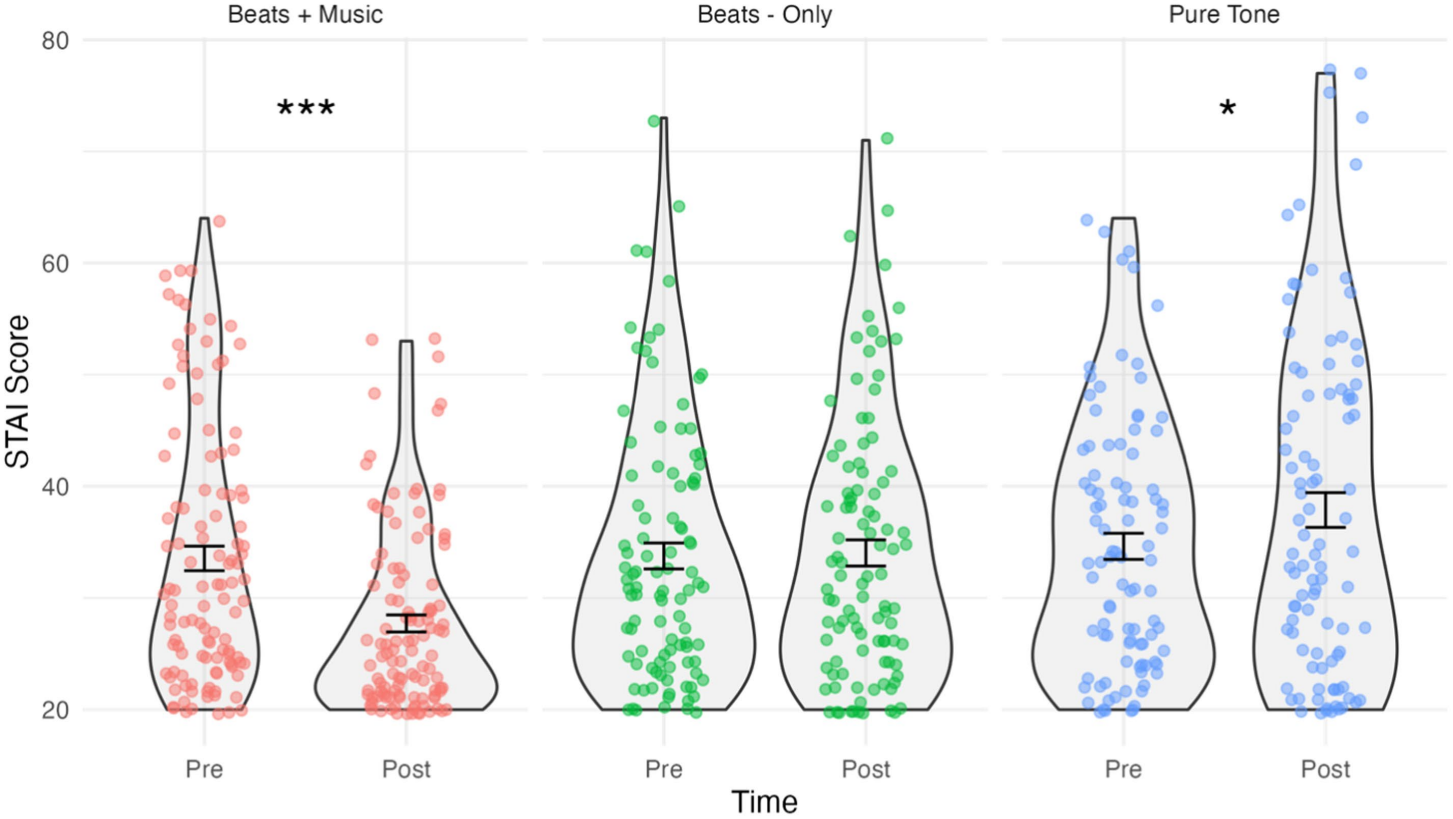
Violin plots showing STAI-S scores by condition and time. Jitterplot shows individual data points. Error bars show means +/− 1 standard error. *p < 0.05, **p < 0.01, ***p < 0.001.

**Figure 2:**
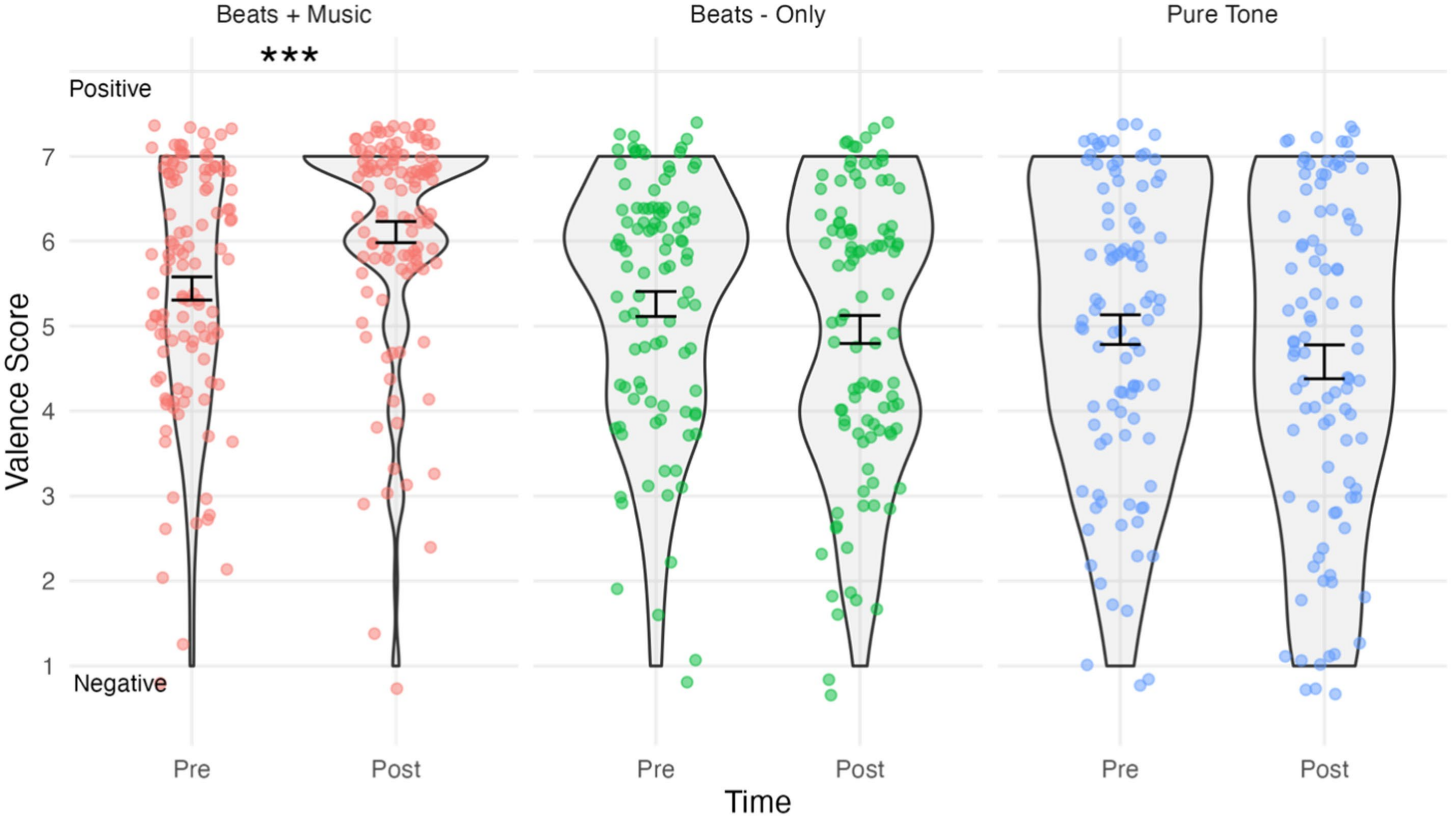
Violin plots showing valence by condition and time. Higher scores indicate “positive” and lower scores indicate “negative” emotional valence. Jitterplot shows individual data points. Error bars show means +/− 1 standard error. ***p < .001.

Three 30-min audio stimuli were created for this study: Beats + Music, Beats-Only, and a Pure Tone Control. The audio exposure duration of 30 min was determined based on Mallik and Russo (2022), which used a 24-min protocol in a similar between-subjects study design. The music track was a 30-min mix of *While We’re Young* by Jhené Aiko, selected for its harmonic and rhythmic stability, as well as pre-testing results indicating that it was broadly perceived as relaxing (see Supplementary Table 1 and Figures 1, 2). Monaural beats were generated to align with the track’s rhythmic and tonal structure. To match the rhythm, a beat frequency of 1.067 Hz was used—corresponding to one cycle per quarter note at the track’s tempo (64 beats per minute). To match the tonality, carrier tones at 219.465 Hz and 220.535 Hz (a 1.067 Hz difference) were used, producing a perceived pitch at 220 Hz (A3), the dominant tone in the track’s key of D major. This tone was harmonically consistent with the track’s chord structure, which primarily comprised Dmaj7 and Gmaj7 (see Supplementary Figure 3). The two carrier tones were set to equal amplitude, producing 100% modulation depth for maximum beat salience. Relative to the music, the beats were embedded at −34.7 decibels relative to full scale (dBFS), which pre-testing determined to correspond to 54.6 dB SPL (A-weighted) when played through headphones at maximum device volume—the average preferred loudness for the Beats-Only stimulus (see Supplementary material, Pre-test 2). A 0.1 dBFS headroom was reserved in the Beats + Music stimulus to prevent clipping or distortion. The Pure Tone Control stimulus consisted of a single tone at 220 Hz, calibrated to 54.6 dB SPL under the same playback conditions.

### Self-reported measures

Participants completed self-report assessments of anxiety and affect immediately before and after each listening intervention. State anxiety was measured using the State subscale of the State–Trait Anxiety Inventory (STAI-S; Spielberger et al., 1983). Mood was assessed using three bipolar 7-point Likert scales: “negative” to “positive” (valence), “tired” to “energetic” (energy), and “sleepy” and “alert” (arousal), following Garrido and Schubert (2013). For all scales, participants were asked to rate how they were feeling at that moment.

### Randomization and blinding

This was a single-blind study: participants were unaware of their condition assignment, while experimenters were aware during data analysis. To maintain participant blinding, details about the study purpose, conditions, and audio stimuli were omitted from the consent form and participant instructions. Accordingly, participants had no knowledge of whether the condition to which they were assigned was “treatment” or “control.” Condition assignment was handled automatically using Qualtrics’ randomization algorithm, which was configured to allocate participants evenly across the three conditions. Because the study was fully automated with no human interaction during task completion, there was no risk of differential experimenter influence on participant responses. Although experimenters were not blinded during data analysis, potential bias is mitigated through transparent reporting of methods and results.

### Procedure

After randomization, participants completed a validated headphone screening test that uses dichotic Huggins pitch stimuli which can only be heard with proper left/right channel separation to confirm headphone use (Milne et al., 2021). This is a standard method for ensuring adequate listening standards and minimizing environmental noise in online psychoacoustic research (e.g., Orpella et al., 2025). Participants who failed the headphone screen—by incorrectly identifying the dichotic pitch on one or more of three trials—were not allowed to continue. The remaining participants were randomly allocated to one of the three experimental conditions. Participants were instructed to set their computer volume to maximum, press play, sit quietly with eyes closed, and avoid movement, reading, or other activities during the 30-min listening session. After the audio concluded, a “next” button appeared, triggering the post-intervention assessments. An attention check was included in post-intervention measures but not during the listening period (Mallik and Russo, 2022).

### Statistical analysis

Analyses were conducted in R using the rstatix and car packages (R Core Team, 2024). A mixed-model repeated-measures ANOVA was performed for each outcome variable (STAI-S, valence, energy, and arousal) with Time (pre vs. post) as a within-subjects factor and Condition (Beats + Music, Beats-Only, or Pure Tone Control) as a between-subjects factor. Assumptions were evaluated by testing the normality of all combinations of time and condition via Shapiro Wilk tests. Effect sizes for the ANOVAs are reported as partial eta squared (*η_p_^2^*). *Post hoc* pairwise comparisons are corrected using the Holm-Bonferroni method, and effect sizes were reported as Cohen’s *d*.

## Results

### Participant demographics

Out of the 450 recruited participants, 135 failed to pass the headphone screener and did not proceed to condition randomization. No data was collected from these participants. An additional 7 participants were excluded after completing the intervention for failing the post-interventions attention checks, leaving a total sample of 308 participants for analysis. A one-way ANOVA showed no significant effect of Condition on Age *F*(2,305) = 0.1, *p* = 1 or Gender *F*(2,304) = 1.44, *p* = 0.238 at baseline. One-way ANOVAs comparing baseline STAI-S, valence, energy, and arousal confirmed that there were no significant differences between the three conditions (see Table 1 for number of participants and demographics by condition and results of one-way “ANOVA”).

**Table 1:** Descriptives of main study participants by condition and effects of condition on emotional arousal, energy, valence and STAI-S.

	Beats + Music *M*(SD)	Beats − Only *M*(SD)	Pure Tone *M*(SD)	*df*	*F*	*p*
*N*	113	100	95	–	–	–
Mean (age)	39.28	38.55	38.81	–	–	–
SD (age)	12.51	11.79	12.38	–	–	–
% Female	51.33	46.00	38.95	–	–	–
STAI-S	33.54 (11.68)	33.76 (11.61)	34.62 (11.39)	305	0.24	0.783
Emotional Valence	5.44 (1.45)	5.26 (1.47)	4.96 (1.7)	305	2.58	0.077
Emotional Energy	5.19 (1.8)	5.32 (1.5)	5.28 (1.58)	305	0.19	1.00
Emotional Arousal	4.68 (1.82)	4.79 (1.5)	4.61 (1.69)	305	0.28	0.754

### Anxiety

The results of the STAI-S analysis are shown in Figure 1. The assumption of normality was met for all combinations of “Time and Condition”, as evidenced by the non-significant Shapiro-Wilks tests (*p* > 0.05 for all groups). The mixed ANOVA predicting STAI-S scores indicated a significant Time*Condition interaction effect, *F*(2,305) = 16.5, *p* < 0.001, *η_p_^2^* = 0.098. Holm–Bonferroni corrected pairwise comparisons showed that STAI-S scores in the Beats + Music condition significantly decreased post-listening (*M* = 27.72, *SD* = 8.11) compared to pre-listening (*M =* 33.54, *SD =* 11.68); *t*(112) *=* 38.55, *p* < 0.001, *d* = –0.58. Cohen’s *d* indicates a medium effect size. In contrast, there was a small, significant increase in STAI-S scores in the Pure Tone Control condition post-listening (*M =* 37.87, *SD =* 15.16) compared to pre-listening (*M =* 34.62, *SD =* 11.31); *t*(94) = 6.42, *p* = 0.028, *d =* 0.24. No significant change in STAI-S scores were observed in the Beats-Only condition post-listening (*M =* 34.03, *SD =* 11.72) versus pre-listening (*M =* 33.76, *SD =* 11.61); *t*(99) = 0.05, *p =* 0.831.

### Affect

The results of the emotional valence analysis are shown in Figure 2. The assumption of normality was met for all combinations of time and conditions, as evidenced by the non-significant Shapiro-Wilks tests (*p* > 0.05 for all groups). The mixed ANOVA predicting valence scores indicated a significant Time*Condition interaction effect, *F*(2,305) = 12.09, *p* < 0.0001, *η_p_^2^ =* 0.073. Holm–Bonferroni corrected pairwise comparisons indicated that valence scores in the Beats + Music condition were significantly higher post-listening (*M =* 6.11, *SD =* 1.33) compared to pre-listening (*M =* 5.44, *SD =* 1.45), *t*(112) = 26.65, *p <* 0.0001, *d* = 0.48, indicating a medium size effect increase in positive affect. No significant changes were observed for valence scores in the Beats-Only condition post-listening (*M =* 4.96, *SD =* 1.65) versus pre-listening (*M =* 5.26, *SD =* 1.47); *t*(99) = 2.71, *p =* 0.134. Similarly, there were no significant changes for valence scores in the Pure Tone Control condition post-listening (*M =* 4.58, *SD =* 1.94) versus pre-listening (*M =* 4.96, *SD =* 1.7); *t*(94) = 3.44, *p =* 0.134.

### Energy

**Figure 3:**
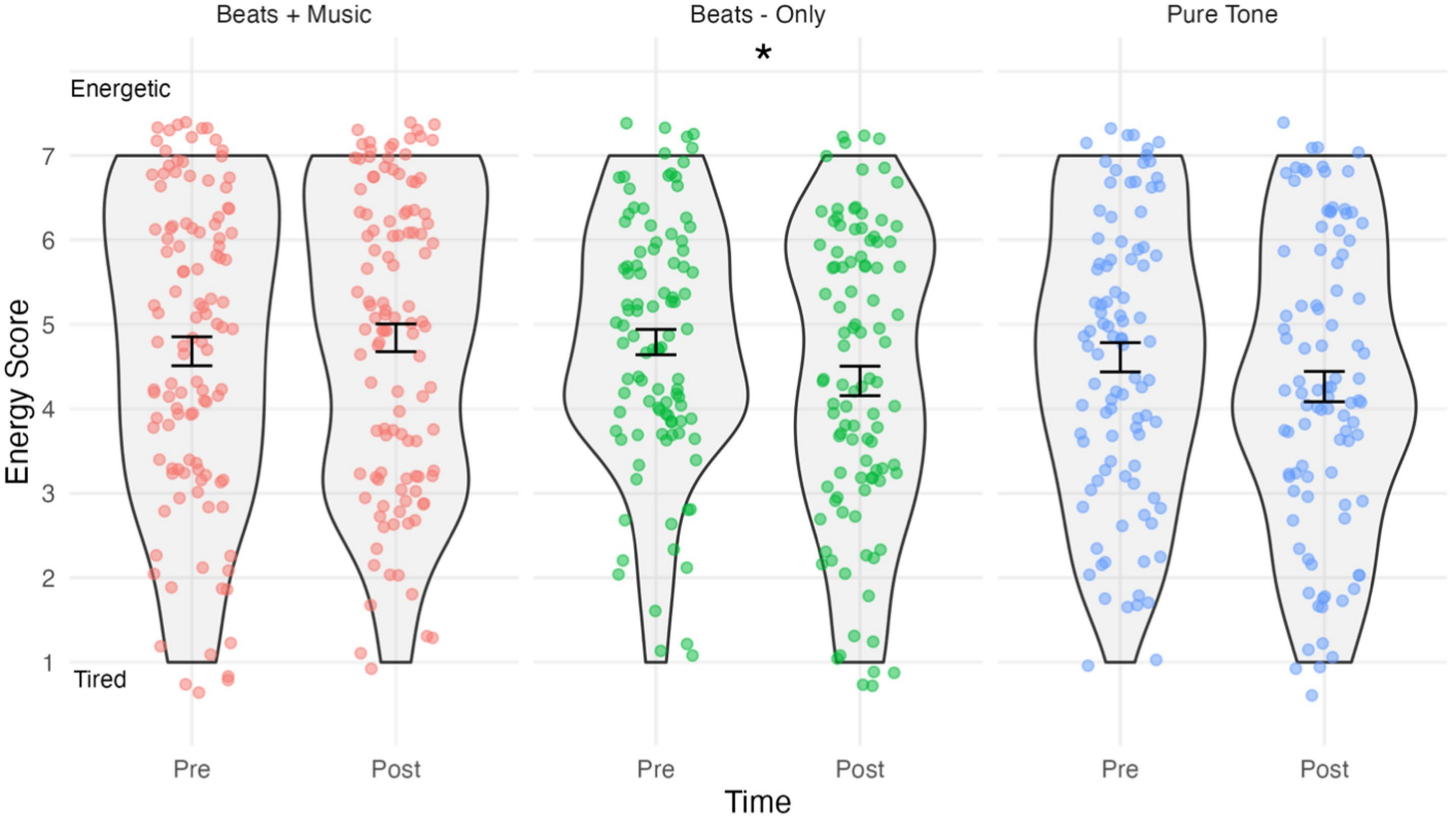
Violin plots showing energy scores by condition and time. Higher scores indicate “energetic” and lower scores indicate “tired”. Jitterplot shows individual data points. Error bars show means +/− 1 standard error. *p <.05.

The results of the energy analysis are shown in Figure 3. The assumption of normality was met for all combinations of time and conditions, as evidenced by the non-significant Shapiro-Wilks tests (*p* > 0.05 for all groups). The mixed ANOVA predicting energy scores indicated a significant Time*Condition interaction effect *F*(2,305) = 4.38, *p <* 0.05, *η_p_^2^* = 0.028. Holm–Bonferroni corrected pairwise comparisons indicated that energy scores in the Beats-Only condition were significantly lower post-listening (*M =* 4.33, SD *=* 1.75) compared to pre-listening (*M =* 4.79, SD *=* 1.50); *t*(99) = 7.35, *p* = 0.024, *d =* −0.28, indicating a small effect sized increase in tiredness. No significant changes were observed in energy scores in the Beats + Music post-listening (*M =* 4.84, *SD =* 1.75) versus pre-listening (*M =* 4.68, *SD =* 1.82); *t*(112) = 1.15, *p =* 0.286. No significant changes were observed in energy scores in the Pure Tone Control condition post-listening (*M =* 4.26, *SD =* 1.75) versus pre-listening (*M =* 4.61, *SD =* 1.75); *t*(94) = 4.28, *p =* 0.082.

### Arousal

The assumption of normality was met for all combinations of time and conditions for the arousal data, as evidenced by the non-significant Shapiro-Wilks tests (*p* ≥ 0.05 for all groups). *F*(1, 305) = 42.5, *p ≤* 0.0001, *η_p_^2^* = 0.12, indicated a general increase in sleepiness across all conditions. There was, however, no significant main effect of Condition, *F*(2, 305) = 0.08, *p* = 0.93, and no significant Time*Condition interaction (2, 305) = 1.38, *p* = 0.25, suggesting no differential effects across experimental conditions. Data is available upon request.

## Discussion

The results revealed significant, medium-sized effects of the Beats + Music condition in reducing self-reported anxiety and enhancing emotional valence, consistent with prior work on auditory beats (e.g., Garcia-Argibay et al., 2019; Mallik and Russo, 2022). Neither the Beats-Only nor Pure Tone Control conditions produced significant effects on anxiety or mood, suggesting that the observed benefits were specific to the combination of beats and music. Notably, the Beats-Only condition showed a small but significant reduction in self-reported energy (i.e., increased tiredness), with a similar trend in the Pure Tone Control, while the Beats + Music condition showed a non-significant trend toward increased energy. These findings support the hypothesis that embedding harmonically and rhythmically congruent monaural beats within music can more effectively reduce anxiety and improve mood than beats alone or comparable non-beating auditory stimuli.

Importantly, these results do not demonstrate that adding monaural beats enhances the anxiolytic or mood-elevating effects of music. Rather, they suggest that such beats can be “embedded” in music without disrupting its well-established therapeutic properties (e.g., Hennessy et al., 2021; Jiang et al., 2013; Stratton and Zalanowski, 1991; Sokhadze, 2007). The absence of significant effects on anxiety or mood for the Beats-Only condition “contrasts” with some prior findings (e.g., Chaieb et al., 2017), potentially reflecting suboptimal stimulation parameters. Supporting this interpretation, participants reported increased tiredness after listening to beats alone for 30 min, which may have dampened any positive effects. It is also possible that this fatigue effect reflects the relatively low frequency of our beat stimulus (1.067 Hz, in the delta range), which is typical of slow wave sleep. Regardless of mechanism, the results indicate that auditory beats exert different effects when presented alone versus embedded in music. This aligns with the findings of Mallik and Russo (2022), who also observed that music combined with beats (this time in the theta range) reduced anxiety, while beats alone did not. Taken together, these results suggest that while the beat frequency for anxiolysis may be somewhat flexible, musical embedding may be important for tolerability and therapeutic efficacy.

Ultimately, identifying which aspects of our stimulation protocol account for discrepancies with previous studies is hindered by substantial methodological variability in the existing literature. Studies differ widely in stimulation “dosage” (duration; range = 180 s – 60 min), beat frequency (range = 1 Hz – 18,000 Hz), center frequency, as well as in beat type (monaural vs. binaural), embedding practices, and experimental context (e.g., peri-operative, at-home use, laboratory testing, passive listening vs. task performance; see Supplementary Table 6). Additional variability stems from stimulation design—for example, while our protocol used a stable beat throughout the session, Chaieb et al. (2017) varied the center frequency over time. Finally, as noted in the introduction, critical acoustic parameters reported in our study (e.g., modulation depth, amplitude relative to embedding stimulus) are often missing from prior work. More consistent and detailed reporting of beat characteristics will be essential for facilitating cross-study comparisons in future research.

### Limitations

Several limitations of this study warrant consideration. First, the headphone exclusion rate was relatively high (29.3%) compared to prior studies (e.g., just 8.2% in Orpella et al., 2025). Given the psychoacoustic nature of our task and our goal of influencing listener psychology, rigorous headphone screening was prioritized. We used a conservative test that typically passes only 80% of true headphone users and may have further increased its difficulty by administering only three trials instead of the recommended six (Milne et al., 2021). Importantly, our screener was administered before randomization to prevent potential allocation bias. Future studies may improve compliance by more strongly emphasizing headphone use in instructions.

Second, using a single musical track limits generalizability. This was an intentional decision aimed at prioritizing validation of core methodological elements—beat-to-music harmonic and rhythmic congruence, monaural beat efficacy, and embedding parameters (modulation depth, relative amplitude). To further advance the method employed here, future studies will need to expand testing to diverse musical contexts. Practical guidance for beat embedding as performed here is provided in the Supplementary material.

Third, the absence of a music-only control prevents us from directly assessing whether the addition of beats enhances the anxiolytic effects of music. While this is distinct from the present study’s focus, it remains an important question for justifying the therapeutic value of combining beats with music.

Finally, the online format limited control over listening conditions (e.g., volume, ambient noise, temperature). While this reduced experimental control, it increased ecological validity and sample diversity—important for real-world applications. Importantly, prior research has shown that auditory beats can produce significant psychological effects in both lab-based and online settings (cf. Chaieb et al., 2017; Mallik and Russo, 2022), suggesting that strict environmental control is not essential. Moreover, because condition assignment was randomized, there is no reason to expect these factors to have varied systematically across groups.

## Conclusion

This study provides evidence that music embedded with rhythmically and harmonically congruent monaural beats can significantly reduce self-reported state anxiety and enhance mood in a non-clinical sample. These effects were not observed for monaural beats presented alone or for a pure-tone control. While this study did not test whether beats enhance the therapeutic effects of music, the results demonstrate that beats can be embedded without diminishing music’s established benefits for anxiety and mood—opening the door to clinical and real-world applications that combine music’s emotional impact with targeted neuromodulation. Moving forward, efforts to replicate and extend these findings across diverse musical contexts, stimulation parameters, and populations will be essential for applying auditory beats in scalable, effective music-based interventions for anxiety and mood regulation.

## Data Availability

The data supporting the conclusions of this article will be made available by the authors, upon reasonable request.

## Footnotes

^1^ www.prolific.com

## References

[ref1] Aarts R. M. Ouweltjes O. Bulut M. (2014). An electro-acoustic implementation of Tibetan bowls: acoustics and perception. Noise Vibrat Worldwide 45, 12–23. doi: 10.1260/0957-4565.45.1.12, PMID: 39154575

[ref2] Bang Y. R. Choi H. Y. Yoon I.-Y. (2019). Minimal effects of binaural auditory beats for subclinical insomnia: a randomized double-blind controlled study. J. Clin. Psychopharmacol. 39, 499–503. doi: 10.1097/JCP.0000000000001097, PMID: 31433343

[ref3] Barratt M. J. Maddox A. Smith N. Davis J. L. Goold L. Winstock A. R.. (2022). Who uses digital drugs? An international survey of ‘binaural beat’ consumers. Drug Alcohol Rev. 41, 1126–1130. doi: 10.1111/dar.13464, PMID: 35353927

[ref4] Bhusari B. N. Hugar S. M. Kohli N. Karmarkar S. Gokhale N. Saxena N. (2023). Comparative evaluation of anxiety level during restorative treatment using no music, monaural beats, and binaural auditory beats as audio distraction behavior guidance technique in children aged 6–12 years: a randomized clinical trial. J. Indian Soc. Pedodont. Prevent. Dentist. 41, 156–162. doi: 10.4103/jisppd.jisppd_104_23 37635475

[ref5] Carvajal M. P. Solano M. A. Herrera-Martínez M. Useche-Ramírez J. E. González J. (2024). Acoustic characterization of sound stimuli from Tibetan singing bowls. Visión Electrón. 18:2. doi: 10.14483/issn.2248-4728

[ref7] Chaieb L. Wilpert E. C. Hoppe C. Axmacher N. Fell J. (2017). The impact of monaural beat stimulation on anxiety and cognition. Front. Hum. Neurosci. 11:251. doi: 10.3389/fnhum.2017.00251, PMID: 28555100 PMC5430051

[ref9] Demirci S. Sezer S. (2024). Effect of binaural beats on anxiety and tolerance in patients undergoing upper gastrointestinal endoscopy without sedation: a randomized controlled trial. J. Integr. Complement. Med. 30, 1209–1216. doi: 10.1089/jicm.2023.0804, PMID: 39088370 PMC11659431

[ref10] Derner M. Chaieb L. Dehnen G. Reber T. P. Borger V. Surges R.. (2021). Auditory beat stimulation modulates memory-related single-neuron activity in the human medial temporal lobe. Brain Sci. 11:364. doi: 10.3390/brainsci11030364, PMID: 33809386 PMC8000797

[ref11] Dos Anjos T. Di Rienzo F. Benoit C.-E. Daligault S. Guillot A. (2024). Brain wave modulation and EEG power changes during auditory beats stimulation. Neuroscience 554, 156–166. doi: 10.1016/j.neuroscience.2024.07.014, PMID: 39004412

[ref12] Dove H. W. (1844). Repertorium der Physik: Enthaltend eine vollständige Zusammenstellung der neuern Fortschritte dieser Wissenschaft. Berlin, Germany: Veit.

[ref13] Draganova R. Ross B. Wollbrink A. Pantev C. (2008). Cortical steady-state responses to central and peripheral auditory beats. Cereb. Cortex 18, 1193–1200. doi: 10.1093/cercor/bhm153, PMID: 17827173

[ref15] Garcia-Argibay M. Santed M. A. Reales J. M. (2019). Efficacy of binaural auditory beats in cognition, anxiety, and pain perception: a meta-analysis. Psychol. Res. 83, 357–372. doi: 10.1007/s00426-018-1066-8, PMID: 30073406

[ref16] Garrido S. Schubert E. (2013). Adaptive and maladaptive attraction to negative emotions in music. Music. Sci. 17, 147–166. doi: 10.1177/1029864913478305, PMID: 40372777

[ref17] Grose J. H. Buss E. Hall J. W. (2012). Binaural beat salience. Hear. Res. 285, 40–45. doi: 10.1016/j.heares.2012.01.012, PMID: 22326292 PMC3299837

[ref18] Hennessy S. Sachs M. Kaplan J. Habibi A. (2021). Music and mood regulation during the early stages of the COVID-19 pandemic. PLoS One 16:e0258027. doi: 10.1371/journal.pone.0258027, PMID: 34669731 PMC8528311

[ref19] Isik B. K. Esen A. Büyükerkmen B. Kilinç A. Menziletoglu D. (2017). Effectiveness of binaural beats in reducing preoperative dental anxiety. Br. J. Oral Maxillofac. Surg. 55, 571–574. doi: 10.1016/j.bjoms.2017.02.014, PMID: 28325532

[ref20] Jiang J. Zhou L. Rickson D. Jiang C. (2013). The effects of sedative and stimulative music on stress reduction depend on music preference. Arts Psychother. 40, 201–205. doi: 10.1016/j.aip.2013.02.002, PMID: 40370555

[ref22] Kelton K. Weaver T. L. Willoughby L. Kaufman D. Santowski A. (2021). The efficacy of binaural beats as a stress-buffering technique. Altern. Ther. Health Med. 27, 28–33, PMID: 32619206

[ref23] Klimesch W. (1999). EEG alpha and theta oscillations reflect cognitive and memory performance: a review and analysis. Brain Res. Rev. 29, 169–195. doi: 10.1016/S0165-0173(98)00056-3, PMID: 10209231

[ref24] Krumhansl C. L. (1997). An exploratory study of musical emotions and psychophysiology. Can. J. Experiment. Psychol. 51, 336–353. doi: 10.1037/1196-1961.51.4.336, PMID: 9606949

[ref25] Kuratomo N. Mashiba Y. Zempo K. Mizutani K. Wakatsuki N. (2019). “Integrating a binaural beat into the soundscape for the alleviation of feelings” in Human-computer interaction–INTERACT 2019. eds. Lamas D. Loizides F. Nacke L. Petrie H. Winckler M. Zaphiris P. (Paphos, Cyprus: Springer International Publishing), 235–242.

[ref26] Kuwada S. Yin T. C. Syka J. Buunen T. J. Wickesberg R. E. (1984). Binaural interaction in low-frequency neurons in inferior colliculus of the cat. IV. Comparison of monaural and binaural response properties. J. Neurophysiol. 51, 1306–1325. doi: 10.1152/jn.1984.51.6.1306, PMID: 6737032

[ref27] Le Scouarnec R. P. Poirier R. M. Owens J. E. Gauthier J. Taylor A. G. Foresman P. A. (2001). Use of binaural beat tapes for treatment of anxiety: a pilot study of tape preference and outcomes. Altern. Ther. Health Med. 7:1. 11191043

[ref28] Loong L. J. Ling K. K. Tai E. L. M. Kueh Y. C. Kuan G. Hussein A. (2022). The effect of binaural beat audio on operative pain and anxiety in cataract surgery under topical Anaesthesia: a randomized controlled trial. Int. J. Environ. Res. Public Health 19:194. doi: 10.3390/ijerph191610194, PMID: 36011825 PMC9408317

[ref29] Mallik A. Russo F. A. (2022). The effects of music & auditory beat stimulation on anxiety: a randomized clinical trial. PLoS One 17:e0259312. doi: 10.1371/journal.pone.0259312, PMID: 35263341 PMC8906590

[ref30] McConnell P. A. Froeliger B. Garland E. L. Ives J. C. Sforzo G. A. (2014). Auditory driving of the autonomic nervous system: listening to theta-frequency binaural beats post-exercise increases parasympathetic activation and sympathetic withdrawal. Front. Psychol. 5:1248. doi: 10.3389/fpsyg.2014.01248, PMID: 25452734 PMC4231835

[ref31] Milne A. E. Bianco R. Poole K. C. Zhao S. Oxenham A. J. Billig A. J.. (2021). An online headphone screening test based on dichotic pitch. Behav. Res. Methods 53, 1551–1562. doi: 10.3758/s13428-020-01514-0, PMID: 33300103 PMC7725427

[ref32] Ölçücü M. T. Yılmaz K. Karamık K. Okuducu Y. Özsoy Ç. Aktaş Y.. (2021). Effects of listening to binaural beats on anxiety levels and pain scores in male patients undergoing cystoscopy and ureteral stent removal: a randomized placebo-controlled trial. J. Endourol. 35, 54–61. doi: 10.1089/end.2020.0353, PMID: 33107329

[ref33] Orpella J. Bowling D. L. Tomaino C. Ripollés P. (2025). Effects of music advertised to support focus on mood and processing speed. PLoS One 20:e0316047. doi: 10.1371/journal.pone.0316047, PMID: 39937723 PMC11819607

[ref34] Oster G. (1973). Auditory beats in the brain. Sci. Am. 229, 94–102. doi: 10.1038/scientificamerican1073-94, PMID: 4727697

[ref35] Padmanabhan R. Hildreth A. J. Laws D. (2005). A prospective, randomised, controlled study examining binaural beat audio and pre-operative anxiety in patients undergoing general anaesthesia for day case surgery*. Anaesthesia 60, 874–877. doi: 10.1111/j.1365-2044.2005.04287.x, PMID: 16115248

[ref36] Parodi A. Fodde P. Pellecchia T. Puntoni M. Fracchia E. Mazzella M. (2021). A randomized controlled study examining a novel binaural beat technique for treatment of preoperative anxiety in a group of women undergoing elective caesarean section. J. Psychosom. Obstet. Gynecol. 42, 147–151. doi: 10.1080/0167482X.2020.1751607, PMID: 32674651

[ref37] Perez H. D. O. Dumas G. Lehmann A. (2020). Binaural beats through the auditory pathway: from brainstem to connectivity patterns. eNeuro 7, ENEURO.0232–ENEU19.2020. doi: 10.1523/ENEURO.0232-19.2020, PMID: 32066611 PMC7082494

[ref38] Pratt H. Starr A. Michalewski H. J. Dimitrijevic A. Bleich N. Mittelman N. (2010). A comparison of auditory evoked potentials to acoustic beats and to binaural beats. Hear. Res. 262, 34–44. doi: 10.1016/j.heares.2010.01.013, PMID: 20123120

[ref39] R Core Team. (2024). R: A Language and environment for statistical computing (Version 4.4.2) [Computer software]. R Foundation for Statistical Computing. Available online at: https://www.R-project.org/ (Accessed October 05, 2024).

[ref40] Salehabadi N. Pakravan A. Rasti R. Pourasghar M. Mousavi S. J. Saravi M. E. (2024). Can binaural beat music be useful as a method to reduce dental patients’ anxiety? Int. Dent. J. 74, 553–558. doi: 10.1016/j.identj.2023.11.009, PMID: 38143164 PMC11123532

[ref41] Sandstrom G. M. Russo F. A. (2010). Music hath charms: the effects of valence and arousal on recovery following an acute stressor. Music Med. 2, 137–143. doi: 10.1177/1943862110371486, PMID: 40372777

[ref42] Schwarz D. W. F. Taylor P. (2005). Human auditory steady state responses to binaural and monaural beats. Clin. Neurophysiol. 116, 658–668. doi: 10.1016/j.clinph.2004.09.014, PMID: 15721080

[ref43] Seetharaman R. Avhad S. Rane J. (2024). Exploring the healing power of singing bowls: an overview of key findings and potential benefits. Explore 20, 39–43. doi: 10.1016/j.explore.2023.07.007, PMID: 37532602

[ref44] Shepherd D. Hautus M. J. Giang E. Landon J. (2023). “The most relaxing song in the world”? A comparative study. Psychol. Music 51, 3–15. doi: 10.1177/03057356221081169, PMID: 40372777

[ref45] Smith C. A. Morris L. W. (1977). Differential effects of stimulative and sedative music on anxiety, concentration, and performance. Psychol. Rep. 41, 1047–1053. doi: 10.2466/pr0.1977.41.3f.1047, PMID: 601132

[ref46] Sokhadze E. M. (2007). Effects of music on the recovery of autonomic and Electrocortical activity after stress induced by aversive visual stimuli. Appl. Psychophysiol. Biofeedback 32, 31–50. doi: 10.1007/s10484-007-9033-y, PMID: 17333313

[ref47] Spielberger C. Gorsuch R. Lushene R. Vagg P. Jacobs G. (1983). Manual for the state-trait anxiety inventory. Palo Alto, CA: Consulting Psychologists Press.

[ref48] Stratton V. N. Zalanowski A. H. (1991). The effects of music and cognition on mood. Psychol. Music 19, 121–127. doi: 10.1177/0305735691192003

[ref49] Stupacher J. Witte M. Hove M. J. Wood G. (2016). Neural entrainment in drum rhythms with silent breaks: evidence from steady-state evoked and event-related potentials. J. Cogn. Neurosci. 28, 1865–1877. doi: 10.1162/jocn_a_01013, PMID: 27458750

[ref50] Stupacher J. Wood G. Witte M. (2017). Synchrony and sympathy: social entrainment with music compared to a metronome. Psychomusicology 27, 158–166. doi: 10.1037/pmu0000181

[ref51] von Helmholtz H. (1863). On the sensations of tone as a physiological basis for the theory of music. Translated by Alexander J. Ellis. Third English edition. London and New York: Longmans, Green, and Co., 1, 6–8.

[ref52] Wahbeh H. Calabrese C. Zwickey H. Zajdel D. (2007). Binaural beat technology in humans: a pilot study to assess neuropsychologic, physiologic, and electroencephalographic effects. J. Alternat. Complement. Med. 13, 199–206. doi: 10.1089/acm.2006.6201, PMID: 17388762

[ref53] Weiland T. J. Jelinek G. A. Macarow K. E. Samartzis P. Brown D. M. Grierson E. M.. (2011). Original sound compositions reduce anxiety in emergency department patients: a randomised controlled trial. Med. J. Aust. 195, 694–698. doi: 10.5694/mja10.10662, PMID: 22171868

[ref54] Wiwatwongwana D. Vichitvejpaisal P. Thaikruea L. Klaphajone J. Tantong A. Wiwatwongwana A.. (2016). The effect of music with and without binaural beat audio on operative anxiety in patients undergoing cataract surgery: a randomized controlled trial. Eye 30, 1407–1414. doi: 10.1038/eye.2016.160, PMID: 27740618 PMC5108018

